# The impact of remission and coexisting migraine on anxiety and depression in cluster headache

**DOI:** 10.1186/s10194-020-01120-7

**Published:** 2020-05-29

**Authors:** Byung-Su Kim, Pil-Wook Chung, Byung-Kun Kim, Mi Ji Lee, Jeong Wook Park, Min Kyung Chu, Jin-Young Ahn, Dae Woong Bae, Tae-Jin Song, Jong-Hee Sohn, Kyungmi Oh, Daeyoung Kim, Jae-Moon Kim, Soo-Kyoung Kim, Yun-Ju Choi, Jae Myun Chung, Heui-Soo Moon, Chin-Sang Chung, Kwang-Yeol Park, Soo-Jin Cho

**Affiliations:** 1 Department of Neurology, Bundang Jesaeng General Hospital, Daejin Medical Center, Seongnam, South Korea; 2 Department of Neurology, Kangbuk Samsung Hospital, Sungkyunkwan University School of Medicine, Seoul, South Korea; 3 Department of Neurology, Eulji Hospital, Eulji University, Seoul, South Korea; 4 Department of Neurology, Neuroscience Center, Samsung Medical Center, Sungkyunkwan University School of Medicine, Seoul, South Korea; 5 Department of Neurology, Uijeongbu St.Mary’s Hospital, The Catholic University of Korea College of Medicine, Uijeongbu, South Korea; 6 Department of Neurology, Severance Hospital, Yonsei University College of Medicine, Seoul, South Korea; 7 Department of Neurology, Seoul Medical Center, Seoul, South Korea; 8 Department of Neurology, College of Medicine, The Catholic University of Korea, Suwon, South Korea; 9 Department of Neurology, Ewha Womans University Seoul Hospital, College of Medicine, Seoul, South Korea; 10 Department of Neurology, Chuncheon Sacred Heart Hospital, Hallym University College of Medicine, Chuncheon, South Korea; 11 Department of Neurology, Korea University College of Medicine, Seoul, South Korea; 12 Department of Neurology, Chungnam National University College of Medicine, Daejeon, South Korea; 13 Department of Neurology, Gyeongsang National University College of Medicine, Jinju, South Korea; 14 Dr. Choi’s Neurology Clinic, Jeonju, South Korea; 15 Department of Neurology, Inje University College of Medicine, Seoul, South Korea; 16 Department of Neurology, Chung-Ang University Hospital, 102 Heukseok-ro, Dongjak-gu, Seoul, 06973 South Korea; 17 Department of Neurology, Dongtan Sacred Heart Hospital, Hallym University College of Medicine, Keun Jae Bong-gil 7, Hwaseong, Gyeonggi-do 18450 South Korea

**Keywords:** Anxiety, Cluster headache, Depression, Headache, Migraine

## Abstract

**Background:**

Our aim was to investigate the relationship between coexisting cluster headache (CH) and migraine with anxiety and depression during active cluster bouts, and how symptoms change during remission.

**Methods:**

We analyzed data from 222 consecutive CH patients and 99 age- and sex-matched controls using a prospective multicenter registry. Anxiety or depression was evaluated using the Generalized Anxiety Disorder-7 (GAD-7) or Patient Health Questionnaire-9 (PHQ-9), respectively. Moderate-to-severe anxiety or depression was defined as a score of ≥10 at baseline (during a cluster bout). We assessed for changes in anxiety and depression during CH remission periods.

**Results:**

Among the CH patients, the prevalence of moderate-to-severe anxiety and depression was seen in 38.2% and 34.6%, respectively. Compared with controls, CH patients were associated with moderate-to-severe anxiety and depression (multivariable-adjusted odds ratio [aOR] = 7.32, 95% confidence intervals [CI] = 3.35–15.99 and aOR = 4.95, 95% CI = 2.32–10.57, respectively). CH patients with migraine were significantly more likely to have moderate-to-severe anxiety and depression (aOR = 32.53, 95% CI = 6.63–159.64 and aOR = 16.88, 95% CI = 4.16–68.38, respectively), compared to controls without migraine. The GAD-7 and PHQ-9 scores were significantly reduced between cluster bout and remission periods (from 6.8 ± 5.6 to 1.6 ± 2.8; *P* < 0.001, and from 6.1 ± 5.0 to 1.8 ± 2.4; *P* < 0.001, respectively).

**Conclusions:**

Our results indicate that CH patients are at increased risk of anxiety and depression, especially in the presence of coexisting migraine. However, the anxiety and depression can improve during remission periods.

## Introduction

Cluster headache (CH) is a rare primary headache disorder characterized by recurrent excruciatingly severe unilateral pain in association with ipsilateral cranial autonomic features [1–3]. The name cluster headache stems from the tendency of the pain attacks to cluster together within bouts that usually last several weeks, which is unlike migraine and any other primary headache disorder. Approximately 80–90% of CH patients have episodic cluster headaches (ECH), where active cluster bouts are separated by remissions lasting more than 3 months.

In primary headaches, mainly migraine, psychiatric comorbidities are important because they can potentially impact the clinical course, quality of life, and headache management negatively [4–8]. Previous studies have shown that CH patients can have psychiatric comorbidities such as anxiety and depression [9–16]. In addition, depressive symptoms can influence disease burden [14]. However, whether the risk of anxiety and depression differs according to the status of the cluster headaches (active bout vs. remission) has not been investigated in detail to date [11, 17, 18]. In addition, in a subset of CH patients ranging from 10.0% to 16.7%, migraine has been reported to coexist [19–22]. Given the well-recognized link between migraine and psychiatric comorbidities, coexisting migraine may independently influence the risk for anxiety and depression in CH patients, although this is a relatively unexplored area to date.

Considering the debilitating nature of repetitive CH attacks during an active cluster bout, we hypothesized that CH patients were at an increased risk for anxiety and depression in active bout period, but that their anxiety and depression would reduce during remission. In addition, given the relationship between migraine and psychiatric comorbidities, we further hypothesized that the risk for anxiety and depression could be influenced by coexisting migraine in CH patients. To test these hypotheses, we conducted a prospective study based on data from a multicenter CH registry to investigate the associations between CH, coexisting migraine, anxiety, and depression during an active episode of cluster bout (acting as the baseline period) and changes in anxiety and depression during remission periods.

## Methods

### Participants and study design

This cross-sectional study was planned as a part of the Korean Cluster Headache Registry (KCHR) study, a prospective, multicenter registry enrolling consecutive CH patients aged ≥19 years at 16 hospitals across Korea - 14 university hospitals (eight tertiary and six secondary referral centers) and 2 secondary referral general hospitals. Patient enrollment was conducted between September 2016 and December 2018 following Institutional Review Board (IRB) approval in each hospital. For the present study, among all the KCHR participants, we enrolled only those with CH compatible with the third edition of The International Classification of Headache Disorders (ICHD-3) [23]. Comorbid migraine was also determined based on the ICHD-3. The detailed protocol of the KCHR has been published elsewhere previously [24–26]. The study protocol was approved by the IRB in each study hospital and complied with the Declaration of Helsinki and Good Clinical Practice guidelines. All patients fully understood the study aims and gave informed written consent before their participation. Due to the nature of our research question, we included patients who enrolled during the cluster bout period of CH.

Control subjects without CH were recruited from 3 out of the 16 hospitals, and were matched on the basis of age and sex. Volunteers aged 19–65 years were invited to participate in this study as a control group, as long as they had no history of diabetes, thyroid illness, severe obesity, severe hepatic or renal illness, or malignancy, and they had the cognitive capacity to complete the questionnaires. Many of the control group participants were friends or relatives of patients with headaches, or employees of the hospital. We evaluated all of the control group participants to determine the presence of migraine based on the ICHD-3. Eligibility of the rest of controls was being headache free (< 1 headache day per month) with no previous history of primary or secondary headache disorder. All control subjects were enrolled after receiving informed written consent.

### Measurement

The KCHR included demographic and clinical data that were collected during the last cluster bout. Anxiety and depression were assessed using the Korean versions of the Generalized Anxiety Disorder 7-item scale (GAD-7) and the Patient Health Questionnaire 9-item scale (PHQ-9). Each item of GAD-7 and PHQ-9 was measured on a four-point scale, from 0 to 3 (0 = never, 1 = several days, 2 = more than half the time, and 3 = nearly every day). Items were scored based on occurrence over the previous 2 weeks. The total scores of GAD-7 and PHQ-9 ranged from 0 to 21 and from 0 to 27, respectively. Based these scores, the patients were classified into three anxiety and depression severity groups: no anxiety (GAD-7 score: 0–4), mild anxiety (GAD-7 score: 5–9), and moderate-to-severe anxiety (GAD-7 score: 10–21), and no depression (PHQ-9 score: 0–4), mild depression (PHQ-9 score: 5–9), and moderate-to-severe depression (PHQ-9 score: 10–27) [18].

Regarding the presence of anxiety, study participants were divided dichotomously into the groups of no anxiety (GAD-7 score ≤ 4) or any anxiety (GAD-7 score ≥ 5) group. The same was done for depression, with the two groups being a no depression group (PHQ-9 score ≤ 4) and an any depression (PHQ-9 score ≥ 5) group. During follow-up, CH patients were asked to repeat the tests of GAD-7 and PHQ-9 when their cluster bout status subsided into remission. Given that GAD-7 and PHQ-9 evaluate the status of anxiety and depression within the last 2 weeks, CH patients performed the follow-up tests of GAD-7 and PHQ-9 after 2 weeks following the end of their cluster bout. During the remission period, we evaluated for a change in GAD-7 and PHQ-9 scores between the cluster bout and the remission period. The exclusion criteria for the remission period analysis were as follows: [1] chronic cluster headache (CCH) [2]; no, or incomplete, response to the GAD-7 or PHQ-9 questionnaire [3]; follow-up GAD-7 and PHQ-9 testing performed within 2 weeks of the end of their cluster bout; and [4] uncertainty as to the end date of the active cluster bout.

### Statistical analysis

To test the study hypothesis, CH patients and control subjects were classified into 4 groups according to the presence of coexisting migraine: a CH with migraine group, a CH without migraine group, a control group with migraine, and a control group without migraine. Continuous variables are presented as mean values ± standard deviation, and categorical variables are presented as numbers (percentage). The Chi-square test for categorical variables and the one-way ANOVA test for continuous variables were used for the assessment of statistical significance of intergroup differences. To identify whether CH and coexisting migraine were associated with anxiety and depression in CH, we performed a logistic regression analysis. The results of the univariate analyses are presented as odds ratios (ORs) accompanied by 95% confidence intervals (CIs). To determine whether CH and coexisting migraine were independent variables contributing to anxiety and depression in CH, we calculated multivariable-adjusted odds ratios (aORs) and 95% CIs after adjusting for potential covariates. To assess for a change in anxiety and depression scores between active cluster bout and remission periods (remission period analysis), we used the paired t-test for the continuous data and the McNemar test for the dichotomous groups we created, as mentioned above. Since we expected a reduction in the GAD-7 and PHQ-9 scores during remission, in those with GAD-7 and/or PHQ-9 scores ≥5 during an acute cluster bout we compared the proportion of participants experiencing a more than 50% reduction in the GAD-7 and PHQ-9 scores, according to prespecified subgroups (age < 40 years vs. age ≥ 40 years, male sex vs. female sex, and presence of migraine vs. lack of migraine). All statistical analyses were performed using SPSS for Windows Version 18.0 (SPSS Inc., Chicago, IL, USA). All *P*-values reported as < 0.05 in two-tailed tests were considered statistically significant.

## Results

### Study participants, anxiety, and depression

**Fig. 1:**
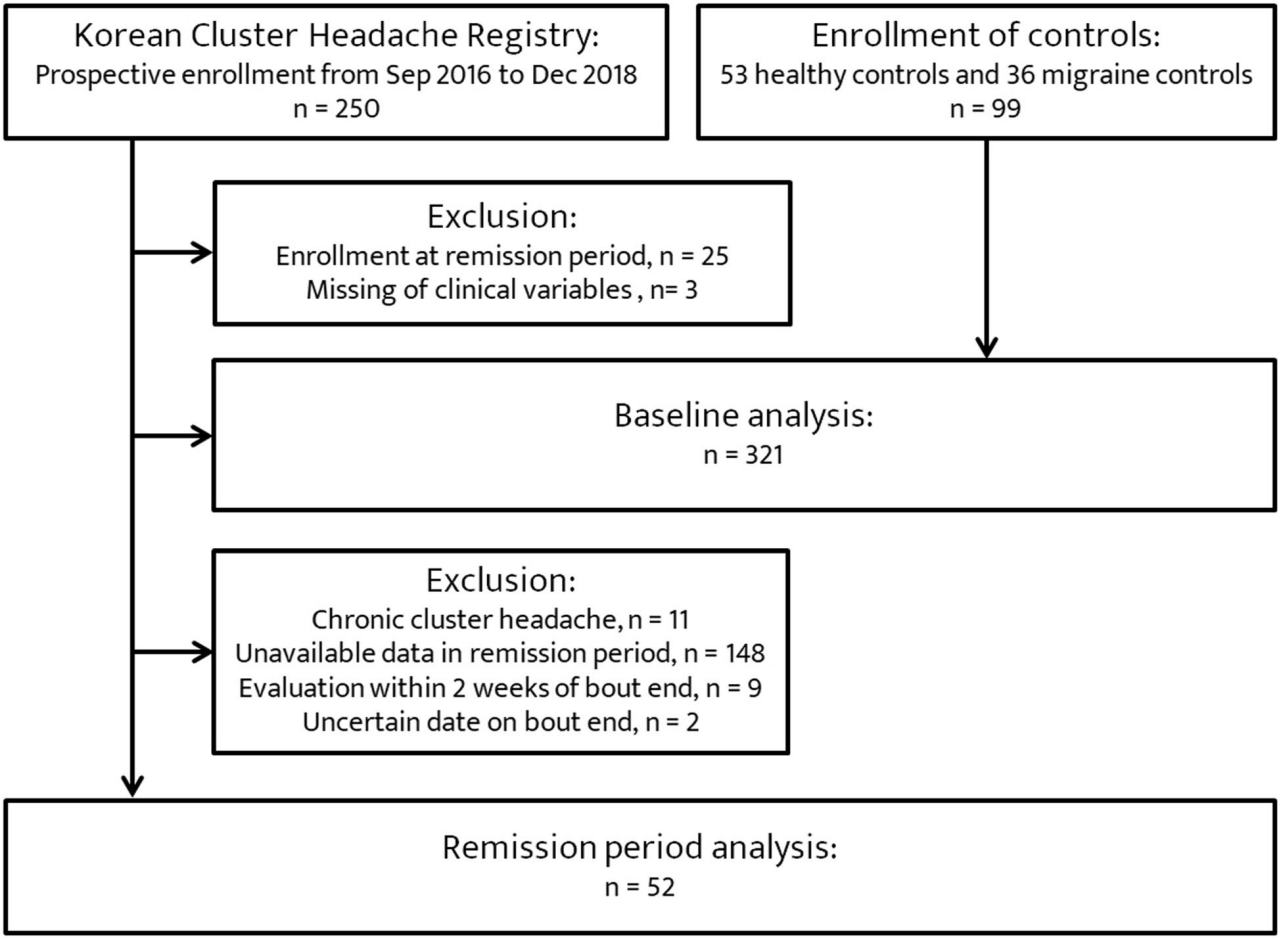
Flowchart for recruitment

During the study period, 222 CH patients and 99 age- and sex-matched controls were enrolled in the multicenter headache registry (Fig. 1). Baseline characteristics between the CH patients and controls are shown in Table 1. No significant differences were observed across the groups of the CH patients and controls in terms of age, sex, current smoking status, and alcohol consumption status. With respect to the CH subtype, of the 222 CH patients, 157 (70.7%), 11 (4.9%), and 24 (10.8%) patients were classified as having ECH (code 3.1.1), CCH (code 3.1.2), and probable CH (code 3.5.1), respectively. For the remaining 33 patients (14.8%), their first episode of CH did not turn into remission within 1 year of their onset or did not follow more than 1 year, so they were classified neither ECH nor CCH, their diagnosis was finally coded as 3.1.

**Table 1:** Baseline characteristics between cluster headache patients and controls

	Control		CH		
	Without migraine	With migraine	Without migraine	With migraine	
Variable	(*n* = 63)	(*n* = 36)	(*n* = 191)	(*n* = 31)	*P*
Age, years	37.6 ± 10.2	34.8 ± 7.4	38.3 ± 11.1	37.2 ± 8.1	0.312
Female sex, n (%)	11 (17.5)	6 (16.7)	26 (13.6)	9 (29.0)	0.188
Current smoking, n (%)	20 (31.7)	13 (36.1)	89 (46.6)	7 (22.6)	0.025
Alcohol drinking, n (%)	42 (66.7)	15 (41.7)	98 (51.3)	10 (32.3)	0.008
GAD-7 score	2.7 ± 3.0	6.2 ± 4.6	7.7 ± 5.5	9.7 ± 6.9	< 0.001
Anxiety, n (%)					< 0.001
None/minimal (GAD-7: 0–4)	50 (79.4)	15 (41.7)	63 (33.0)	9 (29.0)	
Mild (GAD-7: 5–9)	11 (17.5)	13 (36.1)	59 (30.9)	6 (19.4)	
Moderate-to-severe (GAD-7 ≥ 10)	2 (3.2)	8 (22.2)	69 (36.1)	16 (51.6)	
PHQ-9 score	3.1 ± 3.1	6.6 ± 5.2	7.4 ± 6.1	9.4 ± 7.7	< 0.001
Depression, n (%)					< 0.001
None/minimal (PHQ-9: 0–4)	48 (76.2)	17 (47.2)	77 (40.3)	13 (41.9)	
Mild (PHQ-9: 5–9)	12 (19.0)	10 (27.8)	51 (26.7)	4 (12.9)	
Moderate-to-severe (PHQ-9 ≥ 10)	3 (4.8)	9 (25.0)	63 (33.0)	14 (45.2)	

The data are shown as the mean ± standard deviation or number (percentage)

Abbreviations: *CH* Cluster headache; *GAD-7* Generalized Anxiety Disorder (7-item scale); *PHQ-9* Patient Health Questionnaire (9-item scale)

Among the CH patients, the prevalence of anxiety and depression was 67.5% and 59.5%, respectively. The proportion of CH patients with moderate-to-severe anxiety was more than three times that seen in controls (38.2% vs. 10.1%), and the proportion of CH patients with depression was more than double that of controls (34.6% vs. 12.1%). The prevalence of moderate-to-severe anxiety and depression was highest in the CH with migraine group (51.6% and 45.2%, respectively) relative to the CH without migraine group and the two control groups (with and without migraine). Regarding item of suicidal idea in PHQ-9, the CH patients reported any suicidal idea (≥Several days, item score ≥ 1) more frequently, in comparison with the controls (26.1% vs. 10.1%; *P* < 0.001).

### Association of cluster headache and coexisting migraine with anxiety

In univariable analyses, the CH patients were significantly more likely to experience any anxiety and moderate-to-severe anxiety compared to controls (Table 2). Those with coexisting CH and migraine had the highest OR for moderate-to-severe anxiety (OR = 32.53, 95% CI = 6.73–157.12).

**Table 2:** Association of cluster headache and coexisting migraine with anxiety

	Any anxiety (GAD-7 scores ≥5)				Moderate-to-severe anxiety (GAD-7 scores ≥10)			
	Crude OR (95% CI)	*P*	aOR (95% CI)	*P*	Crude OR (95% CI)	*P*	aOR (95% CI)	*P*
Presence of CH^a^								
Control	reference		reference		reference		reference	
CH	3.98 (2.41–6.57)	< 0.001	**5**.17 (2.93–9.12)	< 0.001	5.52 (2.72–11.20)	< 0.001	7.32 (3.35–15.99)	< 0.001
Category of CH and migraine^b^								
Control without migraine	reference		reference		reference		reference	
Control with migraine	5.38 (2.18–13.25)	< 0.001	5.29 (2.10–13.29)	< 0.001	8.71 (1.73–43.71)	0.009	8.43 (1.65–42.96)	0.01
CH without migraine	7.81 (3.95–15.43)	< 0.001	7.71 (3.84–15.49)	< 0.001	17.25 (4.09–72.73)	< 0.001	16.01 (3.77–68.03)	< 0.001
CH with migraine	9.40 (3.50–25.22)	0.001	9.78 (3.55–26.92)	0.001	32.53 (6.73–157.12)	< 0.001	32.53 (6.63–159.64)	< 0.001

^a^Adjustment for age, female sex, current smoking, alcohol drinking, and coexisting migraine

^b^Adjustment for age, female sex, current smoking, and alcohol drinking

Abbreviations: *aOR* Multivariable-adjusted odds ratio; *CH* Cluster headache; *CI* Confidence interval; *GAD-7* Generalized Anxiety Disorder (7-item scale); *OR* Odds ratio

In multivariable analyses, the association between the presence of CH, any anxiety, and moderate-to-severe anxiety remained significant after adjustment for potential covariates. Furthermore, the association between the control with migraine group, the CH without migraine group, and the CH with migraine group and both any anxiety and moderate-to-severe anxiety persisted after multivariable adjustment. The CH with migraine group was associated with an extremely high likelihood of moderate-to-severe anxiety (aOR = 32.53, 95% CI = 6.63–159.64).

### Association of cluster headache and coexisting migraine with depression

In univariable analyses, the presence of CH was significantly associated with any depression and moderate-to-severe depression (Table 3). The CH with migraine group had the highest OR for the presence of moderate-to-severe depression (OR = 16.47, 95% CI = 4.23–64.06).

**Table 3:** Association of cluster headache and coexisting migraine with depression

	Any depression (PHQ-9 scores ≥5)				Moderate-to-severe depression (PHQ-9 scores ≥10)			
	Crude OR (95% CI)	*P*	aOR (95% CI)	*P*	Crude OR (95% CI)	*P*	aOR (95% CI)	*P*
Presence of CH^a^								
Control	reference		reference		reference		reference	
CH	2.80 (1.71–4.59)	< 0.001	2.95 (1.72–5.07)	< 0.001	3.85 (1.98–7.47)	< 0.001	4.95 (2.32–10.57)	< 0.001
Category of CH and migraine^b^								
Control without migraine	reference		reference		reference		reference	
Control with migraine	3.57 (1.49–8.57)	0.004	3.24 (1.31–8.05)	0.011	6.66 (1.67–26.58)	0.007	6.07 (1.45–25.31)	0.013
CH without migraine	4.73 (2.47–9.05)	< 0.001	4.20 (2.16–8.18)	< 0.001	9.84 (2.97–32.62)	< 0.001	8.63 (2.55–29.18)	0.001
CH with migraine	4.43 (1.76–11.11)	0.002	4.07 (1.58–10.52)	0.004	16.47 (4.23–64.06)	< 0.001	16.88 (4.16–68.38)	< 0.001

^a^Adjustment for age, female sex, current smoking, alcohol drinking, and coexisting migraine

^b^Adjustment for age, female sex, current smoking, and alcohol drinking

Abbreviations: *aOR* Multivariable-adjusted odds ratio; *CH* Cluster headache; *CI* Confidence interval; *OR* Odds ratio; *PHQ-9* Patient Health Questionnaire (9-item scale)

In multivariable analyses, the presence of CH had significant associations with any depression and moderate-to-severe depression after adjustment for potential covariates. Using the control without migraine group as a reference, the risk of moderate-to-severe depression was the highest in the group of patients with coexisting CH and migraine (aOR = 16.88, 95% CI = 4.16–68.38). Regarding CH and coexisting migraine, the CH patients with migraine had the biggest odds for suicidal idea of ≥Several days (OR: 8.79, 95% CI: 2.49–30.99; Supplementary Table).

### Change of anxiety and depression between cluster bout and remission periods

Following the predefined exclusion criteria, data on 52 eligible CH patients were finally analyzed in the remission period analysis (Fig. 1). The 52 patients were followed for a median of 87.5 days (interquartile range: 42–131 days) between cluster bout and remission periods. The baseline characteristics of age, female sex, current smoking status, alcohol consumption status, coexisting migraine history, and the GAD-7 and PHQ-9 scores in this group did not significantly differ from those of the other 170 patients excluded from the remission period analysis.

**Fig. 2:**
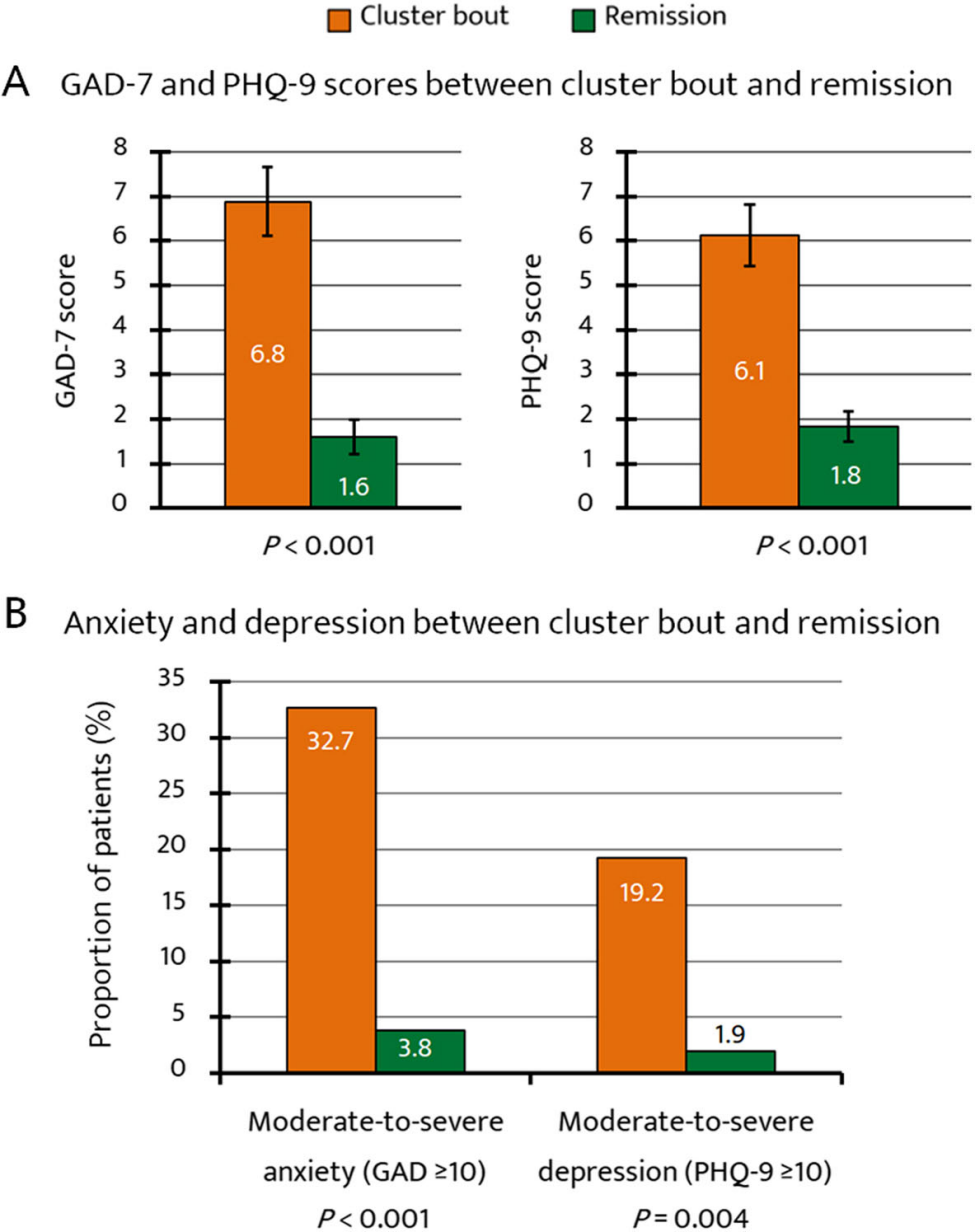
**a** Changes in anxiety and depression scores between active cluster bouts and remission periods. **b** Changes in the proportion of patients with moderate-to-severe anxiety and depression between active cluster bouts and remission periods. Abbreviations: GAD-7, Generalized Anxiety Disorder (7-item scale); PHQ-9, Patient Health Questionnaire (9-item scale)

The mean GAD-7 and PHQ-9 scores reduced significantly between the cluster bout and remission periods (from 6.8 ± 5.6 to 1.6 ± 2.8; *P* < 0.001, and from 6.1 ± 5.0 to 1.8 ± 2.4; *P* < 0.001, respectively; Fig. 2). During the remission period the prevalence of moderate-to-severe anxiety and depression was significantly decreased.

**Fig. 3:**
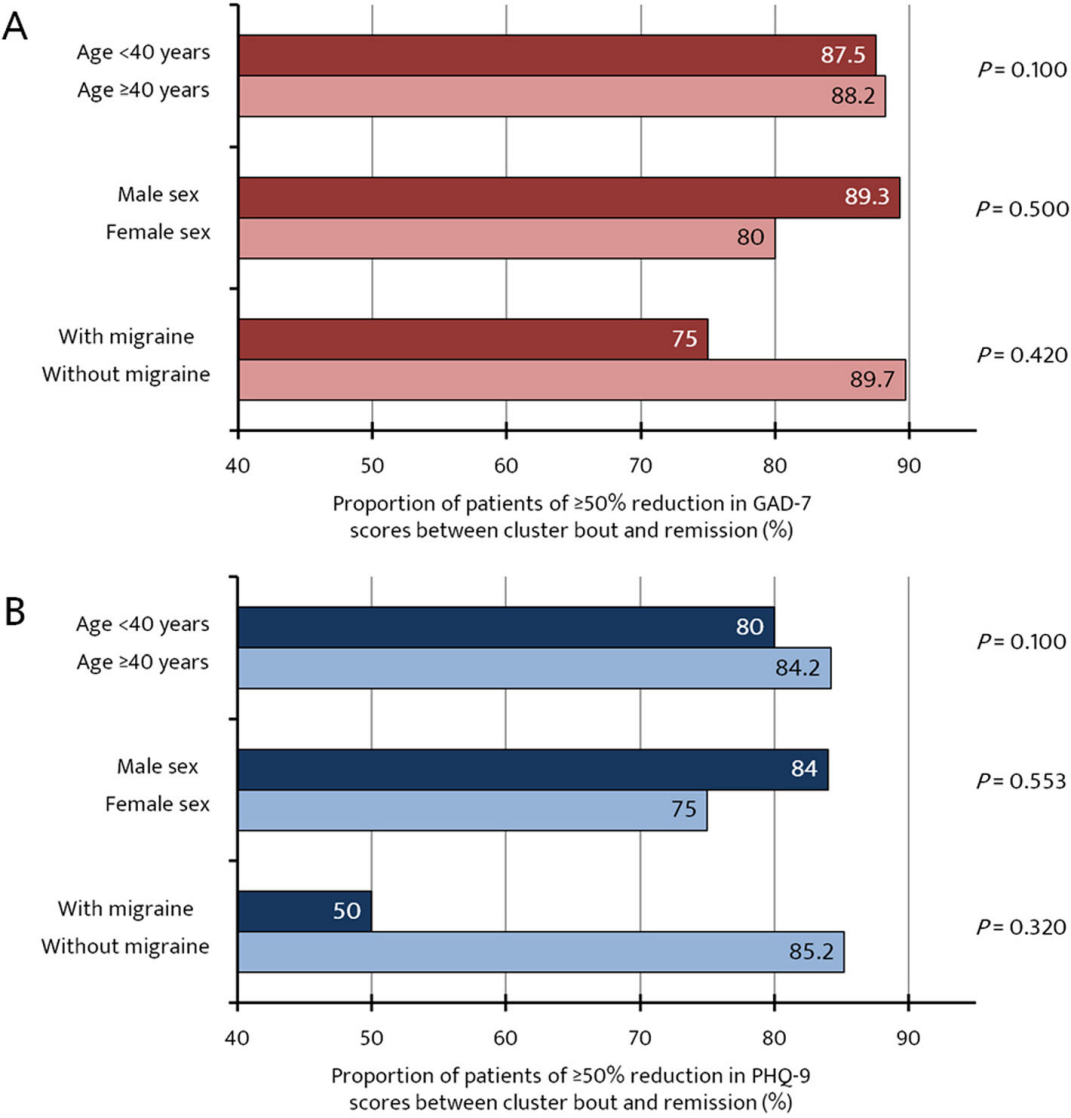
Proportions of patients with a more than 50% reduction in anxiety and depression according to prespecified subgroups. Abbreviations: GAD-7, Generalized Anxiety Disorder (7-item scale); PHQ-9, Patient Health Questionnaire (9-item scale)

The proportions of patients with a more than 50% reduction in GAD-7 and PHQ-9 scores during the remission period were analyzed according to prespecified subgroups (Fig. 3). The proportion of patients with a more than 50% reduction in GAD-7 and PHQ-9 scores was greater in patients without migraine than those with migraine (anxiety: 89.7% vs. 75.0%, and depression: 85.2% vs. 50.0%, respectively); however, the difference did not reach statistical significance.

## Discussion

In the present study, we assessed for an association between CH and coexisting migraine with anxiety and depression, and for any changes between cluster bouts and remission periods. Compared to the control group, the CH patients were significantly more likely to have comorbid anxiety and depression after the multivariable adjustment including coexisting migraine. The associations differed according to the presence of coexisting migraine. Those in the CH with migraine group were at high risk of having moderate-to-severe anxiety and depression. In the remission period following a cluster bout the GAD-7 and PHQ-9 scores were significantly reduced, suggesting that anxiety and depression improve during the CH remission period.

Several early studies showed a wide range in the prevalence of psychiatric comorbidities in CH. The estimated prevalence of anxiety and depression ranged from 11.8% to 75.7% and 6.3% to 43.0%, respectively [9]. These results are insufficient to conclude that CH patients are at an increased risk for having psychiatric comorbidities. Meanwhile, a Taiwanese population study demonstrated that in CH patients without a history of psychiatric disorders, the risk for developing depression was 5.6 times higher than in control subjects [27]. Similarly, a large-scale US study analyzing five-year insurance claims data demonstrated that CH patients, had 2.5 and 2.2 times higher odds of anxiety and depression compared to controls, respectively [12]. In addition, a Dutch cross-sectional study involving 462 CH patients and 177 controls showed that CH patients had nearly double the anxiety and depression scores of control subjects [11]. In line with these findings, the current study can corroborate the notion that there is an increased risk of psychiatric comorbidities in CH patients.

In the present study, it is noteworthy that the CH with migraine group had approximately two-fold higher odds of moderate-to-severe anxiety and depression when compared to the CH without migraine group. This indicates that the bidirectional relationship between migraine and psychiatric comorbidities may further increase the risk of anxiety and depression among CH patients during a cluster bout. Given the debilitating nature of CH, comorbid migraine often comes second place in terms of priority, and receives little or no attention in the clinical management of CH patients during the active cluster bout period. In this context, our findings imply that clinicians should not underestimate the presence and importance of coexisting migraine in the comprehensive and holistic management of CH that incorporates psychiatric well-being.

Despite the strong association between CH, anxiety, and depression during active cluster bouts, anxiety and depression improved remarkably at remission in the present analysis. This suggests that the cyclical nature of CH also applies to the psychiatric comorbidities experienced in CH patients. Prior to our current research, few previous studies had examined this important issue. A German cross-sectional study showed that the proportion of patients with anxiety and depression was higher for patients with ECH at remission than those with ECH during an acute CH episode, which goes against our expectation [17]. A US pilot study conducted a cross-sectional comparison of the levels of anxiety and depression among ECH patients between active cluster bouts and remission periods [18]. The levels of anxiety and depression were similar irrespective of CH status. Like our study, a Dutch study found a strong association between active cluster bout and current depression [11]. However, the association was ultimately attenuated in the final model after adjusting for the scores of sleep disorders. Unlike those studies, our study has an advantage that the findings derive from a prospective observation of the temporal change of anxiety and depression.

We evaluated greater than 50% reductions in the anxiety and depression scale scores according to several prespecified subgroups. Our presumption was that anxiety and depression in female, older patients with coexisting migraine were less likely to improve during the remission period. We observed no significant difference in the subgroup analysis; however, it is interesting to note that the difference in the proportion of patients with more than a 50% reduction in the anxiety and depression scales during remission was greatest between the subgroups of patients with coexisting migraine. This suggests that coexisting migraine has an important influence on the psychological burden of CH patients, and further studies of statistically sufficient sample size are needed to verify this.

Several hypotheses have been already proposed with regard to the risk of affective disorders in CH patients: 1) neuroimaging findings have shown shared anatomical substrates for the pain matrix and depression processing; 2) similarities between CH and depression have been observed in terms of hypothalamic dysfunction and chronobiology; and 3) common mood changes are reported in the pre- and post-ictal phases of a CH attack [11, 28–32]. The possible mechanism underlying the improvement in anxiety and depression during remission periods has not been specifically addressed in the literature to date. Nevertheless, there are some possible explanations. First of all, the termination of the CH attack associated with remission may in itself directly improve the mood of CH patients, under the assumption that mood changes during the pre- and post-ictal phases of a CH attack contribute to the presence of anxiety and depression [32]. Next, nocturnal CH attacks are common during an active cluster bout, which can lead to sleep disturbance as well as depression [11, 21, 31]. In light of this, the lack of nocturnal CH attacks during the remission period may be another reason that improvement in mood is observed. Lastly, neuroimaging studies of CH have reported functional changes in multiple brain networks in relation to cluster bout status [28]. Given the shared anatomical location of the pain matrix and depression processing site, mood improvements may be a consequence of brain changes between active cluster bouts and remission periods.

The present study has several methodological limitations. First, despite the multicenter recruitment of study patients, our results were based on an analysis of data from a hospital-based dataset. In particular, the number of coexisting migraine in the CH patients was resultantly small and similar to the migraine prevalence of general population. Therefore, the findings in the current study require confirmation through additional population-based studies. Second, we estimated the risk of anxiety and depression in CH and coexisting migraine during active cluster bouts based on a cross-sectional analysis. Third, the median follow-up time interval used to assess for a change in anxiety and depression between active cluster bouts and remission was around 3 months. This may be considered short, given that the duration of the remission period varies from months to years. Longer observational studies are required to confirm the presence of dynamic changes in anxiety and depression across repeated phases of active cluster bouts and remission periods. Fourth, the multivariable-adjusted logistic regression analyses to assess the association of CH with anxiety and depression did not adjust for unmeasured potential confounders, such as socioeconomic status, educational level, sleep disorders, more specific physical illness including low back pain, diabetes, and other headache disorders. Further studies complementing this weakness are required to better verify a specific association between CH, coexisting migraine, anxiety, and depression. Fifth, we used the tests of GAD-7 and PHQ-9 to investigate the associations between CH, anxiety, and depression. However, these tests do not confirm the diagnosis of anxiety and depression, but only represent their state, which should be kept in mind. Sixth, due to the moderate sample size of our study, there was a small number of the group of CH and coexisting migraine having anxiety and depression. This may raise a problem throughout the statistical process. In this regard, the results should be interpreted with caution. Finally, the 10.5% patients had probable CH, which might influence the results.

## Conclusions

We have quantified the risk of anxiety and depression in CH and coexisting migraine during active cluster bouts. We have shown that coexisting migraine is a significant influencer on psychiatric comorbidities in patients with CH. Furthermore, anxiety and depression are dynamically altered between active cluster bout and remission periods, suggesting that psychiatric comorbidities may be cyclical in a similar manner to the cluster bouts observed in CH. Further clinical and neuroimaging studies are required to elucidate on the possible mechanisms underlying our findings.

## Supplementary information

**Additional file 1: Table S1.** Association of cluster headache and coexisting migraine with suicidal iedea of Patient Health Questionnaire-9.

## Data Availability

The data used in the present study are available from the corresponding author on reasonable request.

## Abbreviations

aOR: Multivariable-adjusted odds ratio
CCH: Chronic cluster headache
CH: Cluster headache
CI: Confidence interval
ECH: Episodic cluster headaches
GAD-7: Generalized anxiety disorder 7-item scale
ICHD-3: The third edition of the international classification of headache disorders
IRB: Institutional review board
KCHR: Korean cluster headache registry
OR: Odds ratio
PHQ-9: Patient health questionnaire 9-item scale
