# Supplementary material for: The impact of remission and coexisting migraine on anxiety and depression in cluster headache
Source: J Headache Pain. 2020 May 29;21(1):58. doi: 10.1186/s10194-020-01120-7 (PMC7257141; doi:10.1186/s10194-020-01120-7)
Supplement: Supplementary file 1 — Additional file 1: Table S1. Association of cluster headache and coexisting migraine with suicidal iedea of Patient Health Questionnaire-9. [file 10194_2020_1120_MOESM1_ESM.docx]

Supplementary Table. Association of cluster headache and coexisting migraine with suicidal iedea of Patient Health Questionnaire-9.

|  | ≥Several days (score ≥1) | | | |  | ≥Half the days (score ≥2) | | | |
| --- | --- | --- | --- | --- | --- | --- | --- | --- | --- |
|  | Crude OR (95% CI) | *P* | aOR (95% CI) | *P* |  | Crude OR (95% CI) | *P* | aOR (95% CI) | *P* |
| Presence of CH* |  |  |  |  |  |  |  |  |  |
| Control | reference |  | reference |  |  | reference |  | reference |  |
| CH | 3.14 (1.53–6.46) | 0.002 | 3.71 (1.69–8.11) | 0.019 |  | 14.14 (1.89–105.49) | 0.010 | 15.63 (2.01–121.41) | 0.009 |
| Category of CH and migraine† |  |  |  |  |  |  |  |  |  |
| Control without migraine | reference |  | reference |  |  | NA |  | NA |  |
| Control with migraine | 2.95 (0.77–11.25) | 0.113 | 2.50 (0.64–9.80) | 0.187 |  |  |  |  |  |
| CH without migraine | 4.54 (1.56–13.20) | 0.005 | 3.89 (1.31–11.48) | 0.014 |  |  |  |  |  |
| CH with migraine | 10.65 (3.08–36.76) | <0.001 | 8.79 (2.49–30.99) | 0.001 |  |  |  |  |  |

*Adjustment for age, female sex, current smoking, alcohol drinking, and coexisting migraine.

†Adjustment for age, female sex, current smoking, and alcohol drinking.

Abbreviations: aOR, multivariable-adjusted odds ratio; CH, cluster headache; CI, confidence interval; NA, not applicable; OR, odds ratio; PHQ-9, Patient Health Questionnaire (9-item scale).
